# Associations of dietetic care and pregnancy outcomes in women with gestational diabetes

**DOI:** 10.1093/eurpub/ckac131.290

**Published:** 2022-10-25

**Authors:** P van der pligt, G Absalom, J Zinga, C Margerison, G Abbott, S O'Reilly

**Affiliations:** 1 Institute for Physical Activity and Nutrition, Deakin University, Victoria, Australia; 2 School of Exercise and Nutrition Sciences, Deakin University, Victoria, Australia; 3 Department of Nutrition, Royal Women’s Hospital, Victoria, Australia; 4 School of Agriculture and Food Science, University College Dublin, Dublin, Ireland

## Abstract

**Background:**

Gestational diabetes mellitus (GDM) is a significant, global public health problem. Subsequent strain on healthcare systems is widespread and multidisciplinary care may be inadequate. We assessed current nutrition management of GDM in a large, metropolitan maternity hospital in Melbourne, Australia and associations between the model of dietetic care and maternal and neonatal health outcomes.

**Methods:**

Hospital medical record data from The Women’s Hospital, Melbourne for women with GDM (n = 1,185) (July 2105-May 2017) was retrospectively analysed. Adjusted linear and logistic regression were used to assess associations between the number of dietitian consultations and maternal and neonatal health outcomes.

**Results:**

Half of all women received two consultations with a dietitian. Nineteen percent of women received three or more consultations and of these women, almost twice as many were managed by medical nutrition therapy (MNT) and pharmacotherapy (66%) compared with MNT alone (34%). Odds of maternal complications increased with number of consultations (p = 0.008). Lower odds of infant admission to the Neonatal Intensive Care Unit were observed among women receiving one (OR = 0.38 [95% CI: 0.18, 0.78], p = 0.008), two (OR = 0.37 [95% CI: 15 0.19, 0.71], p = 0.003), or three+ dietitian consultations (OR = 0.43 [95% CI: 0.21, 0.88], p = 0.020), compared to no consultations.

**Conclusions:**

The optimal schedule of dietitian consultations for women with GDM in Australia is unclear. Alternative delivery of nutrition education for women with GDM such as telehealth and utilisation of technology may assist in relieving public health and healthcare system pressures and ensure optimal pregnancy outcomes.

**Key messages:**

• Delivering medical nutrition therapy through individual consultations does not deliver a linear benefit to women with GDM and their offspring.

• Alternative delivery modes are needed to optimise outcomes for healthcare services and their patients.

